# A Rare Cause of Secondary Amyloidosis: Common Variable Immunodeficiency Disease

**DOI:** 10.1155/2012/860208

**Published:** 2012-11-14

**Authors:** Ali Kemal Kadiroğlu, Yaşar Yıldırım, Zülfükar Yılmaz, Hasan Kayabaşı, Yahya Avcı, M. Serdar Yıldırım, M. Emin Yılmaz

**Affiliations:** ^1^Department of Nephrology, Faculty of Medicine, Dicle University, 21280 Diyarbakır, Turkey; ^2^Department of Pathology, Faculty of Medicine, Dicle University, 21280 Diyarbakır, Turkey; ^3^Department of Internal Medicine, Faculty of Medicine, Dicle University, 21280 Diyarbakır, Turkey

## Abstract

The common variable immunodeficiency disease (CVID) is the most common symptomatic primary antibody deficiency. It is the most frequently observed cause of panhypogammaglobulinemia in adults. Here, we present a case of systemic amyloidosis that developed secondary to the common variable immunodeficiency disease causing recurrent infections in a young female patient. A 24-year-old female patient, who was under treatment at the gynecology and obstetrics clinic for pelvic inflammatory disease, was referred to our clinic when she was observed to have swellings in her legs, hands, and face. She had proteinuria at a rate of 3.5 gr/day, and her serum albumin was 1.5 gr/dl. The levels of immunoglobulins are IgG: 138 mg/dl, IgA: 22,6 mg/dl, and IgM: 16,8 mg/dl. The renal USG revealed that the kidneys were observed to be enlarged. Since the patient had recurrent infections, hypogammaglobulinemia, nephrotic range proteinuria, and enlarged kidneys in the renal USG, she was thought to have type AA amyloidosis and therefore underwent a renal biopsy. The kidney biopsy revealed amyloid (+). So the patient was diagnosed with AA type of amyloidosis secondary to common variable immunodeficiency disease. A treatment regimen (an ACE inhibitor and a statin) with monthly administration of intravenous immunoglobulin was started.

## 1. Introduction

The common variable immunodeficiency disease (CVID) is the most common symptomatic primary antibody deficiency, and it is the most frequently observed cause of panhypogammaglobulinemia in adults. Leading primarily to chronic or recurrent infections in the lungs and the gastrointestinal system, hypogammaglobulinemia is characterized by normal or diminished numbers of B cells and disturbed antibody response [1, 2]. Secondary amyloidosis is a rather rare complication of the CVID, and the condition has been reported mostly in middle-aged men [3–6]. Generally, in patients where amyloidosis has developed secondary to the CVID, there is an underlying condition such as severe infectious disease, cor pulmonale, congestive hepatomegaly, bilateral bronchiectasis, severe respiratory distress, or tuberculosis [5, 6]. However, a delayed diagnosis are insufficient intravenous immunoglobulin (IVIG) treatment are also factors contributing to the development of amyloidosis [6]. In this paper, our aim is to present a case of systemic amyloidosis that developed secondary to the common variable immunodeficiency disease in a young female patient.

## 2. The Case

A 24-year-old female patient, who was under treatment at the gynecology and obstetrics clinic for pelvic inflammatory disease, was referred to our clinic for further evaluation and treatment when she was observed to have swellings in her legs, hands, and face. She had proteinuria at a rate of 3.5 gr/day in 24 hours, and her serum albumin was 1.5 gr/dL. The patient history revealed that she was frequently ill in her childhood, had recurring episodes of flu during the winter months, and had to be hospitalised due to pneumonia at least once every year. She also suffered from chronic respiratory distress and frequent oral herpes. She had meningitis two years ago, vaginal abscess a year ago, an appendectomy and tuboovarian abscess drainage 6 months ago. In her physical examination, her blood pressure was 90/60 mmHg, both conjunctivae were pale, and pretibial edema was (++/++). Her respiratory system was normal, and she had no organomegaly. The patient's complete blood and biochemistry values on her admission to and discharge from the nephrology clinic are presented in Table 1, and her glomerular filtration rate, proteinuria per day, and urine test results are summarised in Table 2.

The other laboratory tests of the patient revealed 10–15 leukocytes, 3–5 erythrocytes, 6-7 epithelial cells in the urine examination, and no bacterial growth in the urine culture. No light chain excretion was detected in the urinary immune electrophoresis. The results of the other tests were as follows: C3: 83 mg/dL, C4: 37 mg/dL, sedimentation: 92 mm/h, CRP: 0.833 mg/dL, ANA (−), IgG: 138 mg/dL (700–1600), IgA: 22,6 mg/dL (70–400), and IgM: 16,8 mg/dL (40–140); hepatitis panel: HbsAg (−), anti-HbsAg (−), anti-HCV (−), and HIV (−).

**Figure 1:**
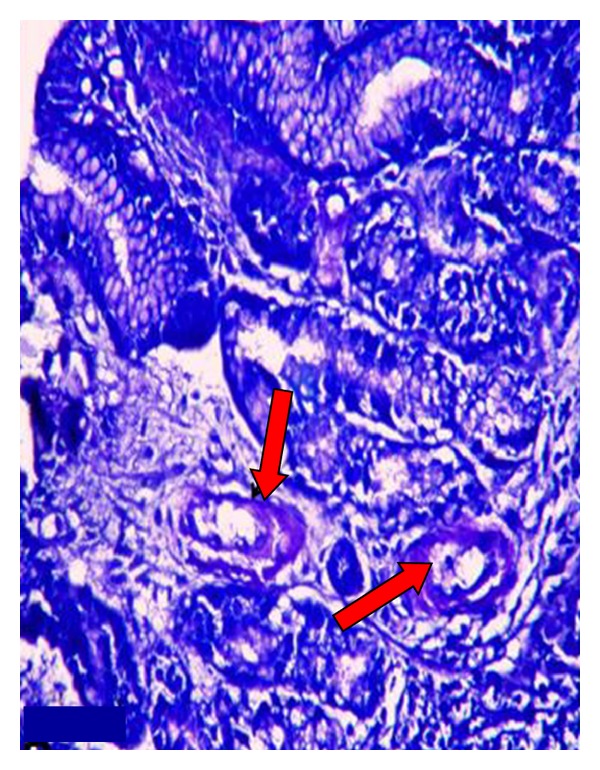
Deposition of amyloid in the walls of blood vessels in lamina propria of duodenum.

Since the patient complained of dyspepsia, an endoscopy was performed together with a duodenal biopsy (Figure 1). According to the endoscopic findings, the mucosa of the bulbus and the second part of the duodenum were observed to be edematous and nodular.

The abdominal and pelvic USG revealed that the liver was in a normal size with a homogenous parenchyma. The gallbladder was observed to be of normal size with sludge inside. The spleen was also of normal size, and the parenchymal echo was homogenous. Both kidneys were observed to be enlarged (right kidney: 140 mm and left kidney: 150 mm), and the parenchymal echo was increased to grade two.

**Figure 2:**
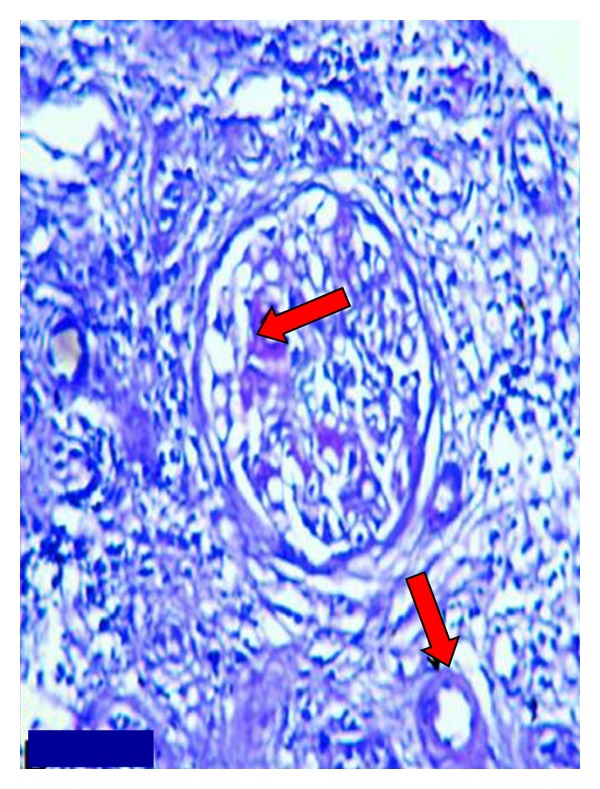
Deposition of amyloid in the focal areas and capillary walls of glomerulus (crystal violet stain ×400).

Since the patient had recurrent infections, hypogammaglobulinemia, hypotension, nephrotic level proteinuria, and enlarged kidneys in the renal USG, she was thought to have type AA amyloidosis and therefore underwent a renal biopsy (Figure 2). In the meantime, the patient was started on a treatment with ACE inhibitors against the proteinuria. The renal biopsy cross-sections containing a total of 12 glomeruli revealed global sclerosis in 2 glomeruli, periglomerular fibrosis in 4 glomeruli, and the buildup of a pale and hyaline-like eosinophilic substance around the papillary loops in all the glomeruli, together with a thickening in the basal membranes. In the interstitium, mononuclear inflammation focussing on the focal perivascular areas was observed. Occasional neutrophil leukocytes were accompanying the inflammation in one area. In the histochemical study with Masson's trichrome stain, concentric lamellar fibrosis around some glomeruli was observed and fibrosis was evident in certain areas of the interstitium. No obvious crescent formation or tubular pathologies were detected using the PAS stain. The crystal violet stain revealed focal amyloid buildup around the capillary loops in all the glomeruli. With the immunofluorescence technique, the IgG, IgA, IgM, C3, C1q, fibrinogen, kappa, and lambda stains revealed no glomeruli, and thus no significant results were obtained.

Since the kidney tissue was amyloid (+) and the duodenal biopsy was observed to be AA amyloid with amyloid buildup in the histochemical study with crystal violet and Kongo red stains, the patient was diagnosed with AA type of amyloidosis secondary to common variable immunodeficiency disease causing recurrent infections in the patient. Hematology consultation was sought for the common variable immunodeficiency disease, and the diagnosis was confirmed. A treatment regimen with monthly administrations of intravenous immunoglobulin was recommended. Statins were added to the patient's current treatment, and she was discharged to return for followup.

## 3. Discussion

The structural and functional changes caused by the extracellular buildup of a special kind of fibrillar protein in various organs are called amyloidosis. The condition caused by the accumulation of the amyloid in the kidneys is defined as renal amyloidosis. Renal amyloidosis may be primary or secondary. Primary renal amyloidosis, where the immunoglobulin that builds up is a light chain, is called AL amyloidosis. In secondary renal amyloidosis, the buildup consists of serum amyloid A (SAA) which is an acute phase protein synthesised in the liver, and this condition is called AA amyloidosis. In this case, the SAA that is in the form of a *β*-folding and is produced in connection with chronic and recurrent infections accumulates extracellularly and leads to damage [7, 8].

The patient was a 24-year-old female, whose patient history revealed that she was frequently ill in her childhood, had recurring episodes of flu during the winter months, and had to be hospitalised due to pneumonia at least once every year. She suffered from chronic respiratory distress and frequent oral herpes. She had meningitis two years ago, vaginal abscess a year ago, an appendectomy and tuboovarian abscess drainage 6 months ago. While she was under treatment for pelvic inflammatory disease, she was observed to have swellings in her legs, hands, and face. She also had proteinuria at a rate of 3.5 gr/day (4.5 gr/day later in the nephrology clinic) within 24 hours and 1.5 gr/dL of serum albumin. Her blood pressure was 90/60 mmHg; her conjunctivae were pale, and she had pretibial edema (++/++). Since the prediagnosis was amyloidosis due to recurrent infections caused by immunodeficiency, a renal biopsy was performed. Meanwhile, the patient was started on antiproteinuric treatment with ACE inhibitors and antilipidemic treatment with a statin. Since she also had complaints related to dyspepsia, gastric involvement of amyloidosis was considered and the patient underwent an endoscopy with duodenal biopsy [9]. Both biopsy samples were reported as amyloid (+) tissue (Figures 1 and 2).

The clinical features of amyloidosis vary according to the involved organ. The most commonly affected organs are kidneys, followed by the gastrointestinal system, joints, thyroid tissue, and gums. While the condition causes malabsorption, perforations, haemorrhage, or obstructions in the gastrointestinal system, it leads to arthropathies in the joints, thyromegaly in the thyroid, and hypertrophy in the gums [10, 11].

Our patient had nephrotic level proteinuria due to kidney involvement, hypoalbuminemia, pretibial edema, and hypotension, as well as dyspeptic complaints due to gastrointestinal involvement. Although urine sediments of the patients with CVID and AA amyloidosis may contain a greater number of erythrocytes compared to the AL type of amyloidosis, the repeated urine tests of our patient did not reveal any erythrocytes. Also, since patients with the common variable immunodeficiency disease generally have asymptomatic proteinuria, it is possible that the connection between the proteinuria and amyloidosis is overlooked during the course of the underlying disease(s), which may be an important cause leading to a belated diagnosis [12].

On the other hand, when the duration of the AA amyloidosis that develops secondary to the CVID is considered, it is observed that the inflammation continues for an average of 8–14 years before the amyloidosis is diagnosed. Thus, a number of infectious episodes that had occurred since the childhood and the inefficient treatment of these infections lead to the development of AA amyloidosis [12]. Our patient expressed that she was frequently ill in her childhood, had recurring episodes of flu during the winter months, had to be hospitalised due to pneumonia at least once every year, and suffered from chronic respiratory distress and frequent oral herpes. It is also known that she had meningitis two years ago, vaginal abscess a year ago, and an appendectomy and tuboovarian abscess drainage 6 months ago. When the age of our patient is considered, it is understood that, although she is at a young age, she has completed the necessary time for the development of renal amyloidosis.

In order to prevent that the patients with common variable immunodeficiency disease are exposed to continuous infectious episodes or that the underlying inflammatory disease leads to AA amyloidosis, they should receive intravenous IVIG replacement treatment without delay. Treatment with IVIG shall dramatically decrease the recurrent infectious episodes and thus the development of systemic amyloidosis.

In conclusion, in patients with a history of frequent infections, hypotension, and nephrotic level proteinuria, systemic amyloidosis secondary to the rare clinical condition of common variable immunodeficiency disease must be considered.

## Figures and Tables

**Table 1:** The patient's admittance and discharge biochemistry and complete blood values.

Parameters	Glucose	Urea	Cre	Na	K	Ca	P	T.pro	Albu.	TRG	Col	Leuko.	Hgb	Platelets
Admittance	76	21	0.6	140	4.1	6.7	4.3	4.0	1.5	320	276	11.4	9.0	445
Discharge	81	45	1.1	140	3.8	8.3	4.4	4.4	2.0	246	208	6.10	10.5	298

**Table 2:** The patient's GFR, proteinuria per day, and complete urine test results.

Parameters	GFR mL/min.	Proteinuria g/day	Density	pH	Protein spot	Microscopy
Admittance	50	4.5	1005	6.5	+++	Ery(−), leuk(++)
Discharge	35	3.5	1006	6.5	+++	Ery(−), leuk(−)
